# Superior Mesenteric Artery Syndrome: A Rare Mimic of Common Causes of Upper Gastrointestinal Obstruction

**DOI:** 10.5005/jp-journals-10018-1098

**Published:** 2014-01-22

**Authors:** Gayatri Madhab, Jette Madsen, Eva Brems Dalgaard, Arindam Bharadwaz

**Affiliations:** 1 Department of Radiology, Viborg Regional Hospital, Viborg, Denmark; 2 Department of Radiology, Viborg Regional Hospital, Viborg, Denmark; 3 Department of Radiology, Viborg Regional Hospital, Viborg, Denmark; 4 Department of Radiology, Aarhus University Hospital, Aarhus, Denmark

**Keywords:** Superior mesenteric artery syndrome, Upper GI obstruction, MDCT, Aortomesenteric angle, Aortomesenteric distance.

## Abstract

Rare causes of common symptoms often pose a diagnostic dilemma which can lead to unwanted loss of precious time in proper diagnosis and management. Superior mesenteric artery (SMA) syndrome is such a rare disorder. It typically presents with signs and symptoms of upper gastrointestinal (GI) obstruction which can be mimicked by a variety of clinical conditions, such as peptic ulcer, chronic pancreatitis, mesenteric ischemia and systemic lupus erythematosus (SLE). A high degree of clinical suspicion is required to direct proper investigations to narrow down to the correct diagnosis. Multi-detector CT (MDCT) proves to be invaluable not only in ruling out more common conditions mimicking SMA syndrome but also in arriving at the correct diagnosis quickly and effectively with great clinical accuracy and thereby removing confusion and delay in proper management.

**Abbreviations:**

SMA: Superior mesenteric artery; GI: Gastrointestinal; MDCT: Multidetector computed tomography; MPR: Multiplanar reconstruction; AMA: Aortomesenteric Angle; AMD: Aortomesenteric distance.

**How to cite this article:** Madhab G, Madsen J, Dalgaard EB, Bharadwaz A. Superior Mesenteric Artery Syndrome: A Rare Mimic of Common Causes of Upper Gastrointestinal Obstruction. Euroasian J Hepato-Gastroenterol 2014;4(1):58-60.

## CASE REPORT

**Fig. 1:**
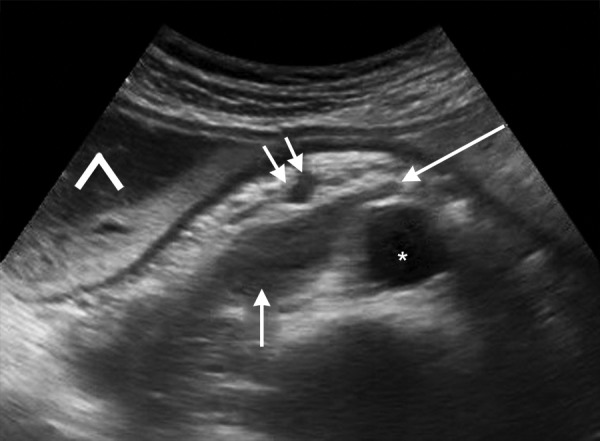
A 61-year-old male presented with symptoms of upper GI obstruction. Ultrasound of abdomen shows distension of stomach (^A^) and duodenum (short single arrow) due to compression of 3rd part of duodenum (long arrow) between SMA (double arrows) and aorta (*)

**Fig. 2:**
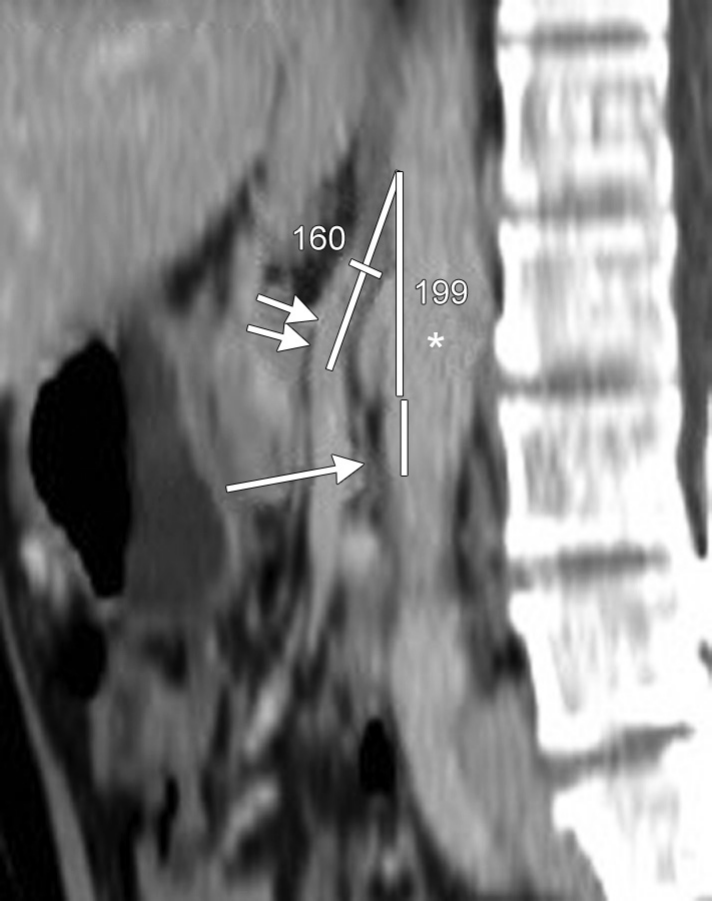
Sagittal reconstructed CT scanning of the same patient as Figure 1 shows narrowed AMA of about 20° between SMA (double arrows) and aorta (*). Narrowed 3rd part of duodenum is seen (long arrow)

**Fig. 3:**
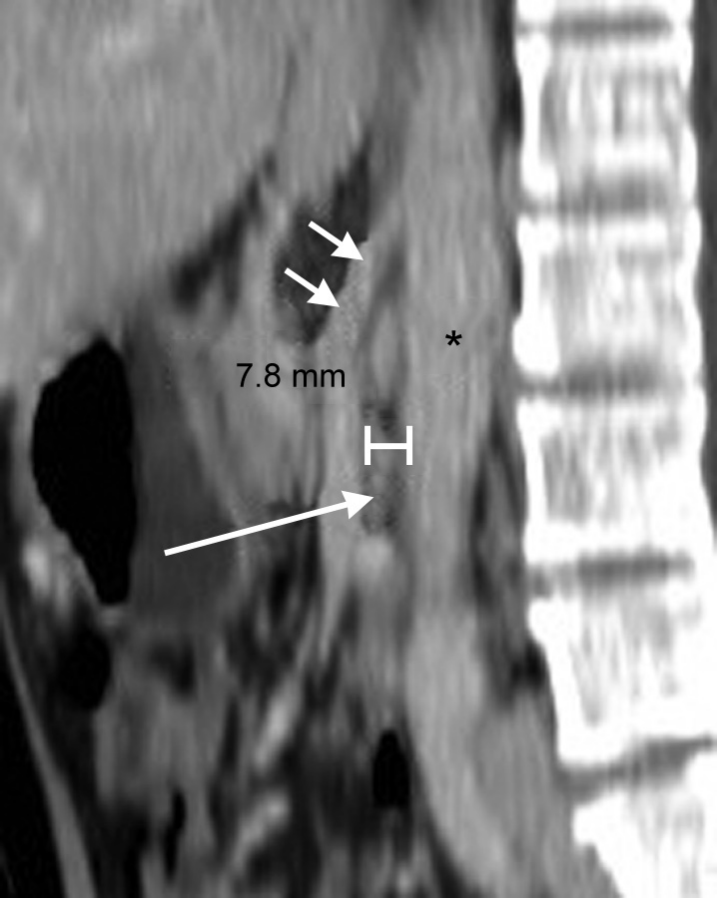
Sagittal reconstructed CT scanning of the same patient as Figure 1 shows reduced AMD of about 8 mm between SMA (double arrows) and aorta (*). Narrowed 3rd part of duodenum is seen (long arrow)

**Fig. 4:**
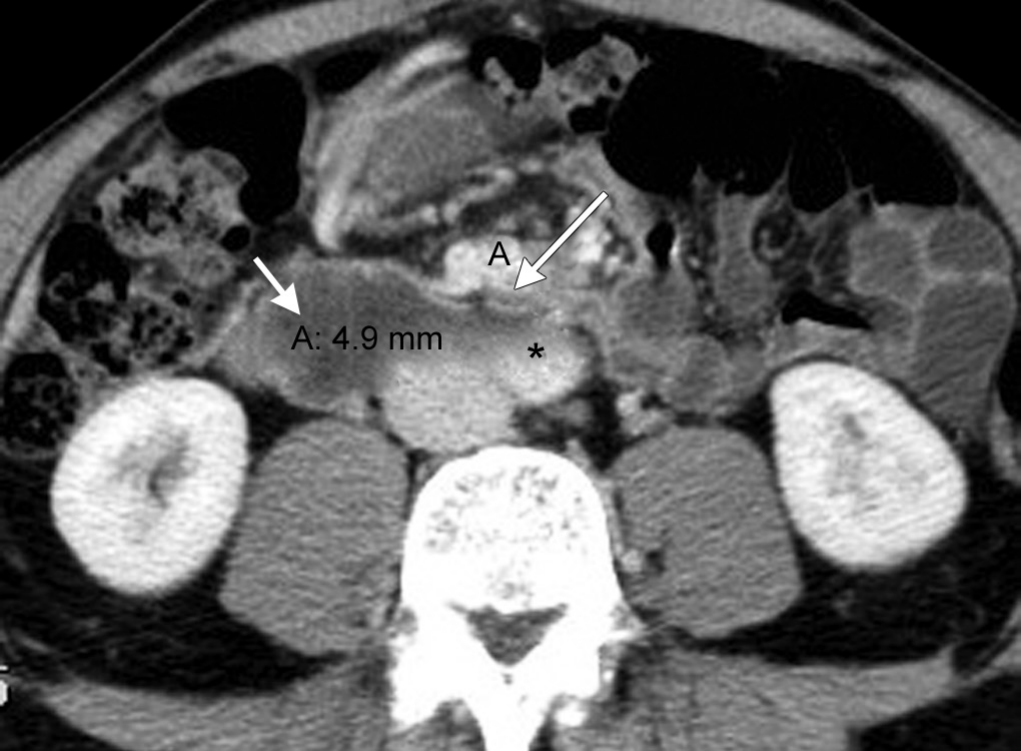
Axial CT scanning of the same patient shows markedly reduced AMD of about 5 mm between SMA (long arrow) and aorta (*) with distension of proximal duodenum (short arrow)

A 61-year-old man presented with symptoms of upper gastrointestinal (GI) obstruction, such as postprandial upper abdominal pain, vomiting and weight loss. He was operated for a perforated duodenal ulcer 12 years back. On physical examination, there was mild tenderness over epigastrium. Blood examinations were normal. Typical imaging findings of superior mesenteric artery (SMA) syndrome were demonstrated. Ultrasound examination revealed gastric and duodenal dilatation (Fig. 1). Multidetector computed tomography (MDCT) with multiplanar reconstruction (MPR) revealed aortomesenteric angle (AMA) of <20° and aortomesenteric distance (AMD) from 5 to 8 mm. There was abrupt narrowing of 3rd part of duodenum with dilatation of stomach and proximal duodenum (up to 2nd part) (Figs 2 to 4). Endoscopy revealed two minor benign peptic ulcers but no other cause of duodenal obstruction. Diagnosis of SMA syndrome was thus made in presence of typical clinical features and classical imaging findings. Patient responded to conservative treatment.

## DISCUSSION

### Etiology and Demographics

Superior mesenteric artery syndrome is a rare disorder where the third part of duodenum is compressed in an abnormally narrow space between aorta and the SMA resulting in symptoms of upper GI obstruction. Its exact incidence is very difficult to measure but estimated to be about 0.1 to 0.3%.^1^ Rokitansky first reported it in 1861, which was later described in detail by Wilkie in 1927.^2,3^ Causes of reduced AMA and AMD leading to SMA syndrome include short ligament of Trietz, low origin of the SMA, rapid adolescent growth, etc. But, more common causes are weight loss due to eating disorders, cancer, postspinal and GI surgery or other chronic debilitating diseases, which result in reduction of the protective fat-pad and lymphatic tissue between the aorta and the SMA causing compression of the duodenum between aorta and SMA.^4,5^ In our case, previous operation for duodenal ulcer could have resulted in disruption of the mesenterial pad of fat leading to the SMA syndrome.

The condition has traditionally been a diagnosis of exclusion where other causes of upper GI obstruction have been ruled out.^6,7^ However, newer modalities, especially MDCT has vastly contributed to its correct and early diagnosis and management.

### Imaging Findings

Imaging studies either directly depict narrowed AMA, reduced AMD, duodenal compression or gastroduodenal distension. Plain and contrast radiography may show gastric and duodenal distension up to compressed part.^8^ Endoscopy may show a pulsatile external compression^5^ and rule out neoplasm and peptic disorders.^9,10^ Ultrasound can be used to measure AMA.^7^ Angiography was previously considered as standard diagnostic modality.^5^ However, MDCT with 3D and MPR is now considered the gold standard by many as it accurately shows abrupt narrowing and obstruction of the third part of the duodenum due to compression by SMA resulting in distension of the stomach and proximal duodenum, helps precisely to measure AMA and AMD, demonstrate compression of the left the renal vein leading to renal vein thrombosis (nutcracker phenomenon), assess intra-abdominal and retroperitoneal fat and rule out other causes, such as pancreatitis and tumor as the cause of duodenal obstruc-tion.^7,11^ Though computed tomography (CT) findings are highly suggestive, they can be found in normal subjects as well and, therefore, needs to be interpreted in the context of individual patient’s clinical symptoms.^12^ An AMA <22° (normal 28-65°) and an AMD <8 mm (normal 10-34 mm) with relevant clinical symptoms are suggestive of SMA syndrome.^9,11,13-15^ A sagittal parallelism between aorta and SMA has also been suggested to be a critical factor.^9,16^

### Differential Diagnosis

Clinical features of SMA syndrome can be found in more common conditions, such as peptic ulcer, pancreatitis and mesenteric ischemia.^17^ They can also be mimicked by SMA-like syndromes, such as scleroderma, diabetes and SLE.^18-20^

## CONCLUSION

Awareness of and a high degree of suspicion for SMA syndrome is needed to direct proper diagnostic investigation. Contrast enhanced MDCT can accurately show obstruction of the third part of the duodenum, help precisely to measure AMA and AMD and rule out other causes of duodenal obstruction thereby contributing to early diagnosis and proper management of SMA syndrome.
